# Les tumeurs conjonctives cutanées: à propos de 121 cas

**DOI:** 10.11604/pamj.2014.17.144.3945

**Published:** 2014-02-28

**Authors:** Fatima Ezzahra Hazmiri, Anas Fakhri, Hanane Rais, Nadia Akhdari, Said Amal, Badia Belaabidia

**Affiliations:** 1 Service d'anatomie pathologique, CHU Mohammed VI Marrakech, Maroc; 2 Service de dermatologie, CHU Mohammed VI Marrakech, Maroc

**Keywords:** Tumeur conjonctive cutanée, epidémiologie, anatomie pathologique, evolution, Conjunctiva skin tumor, epidemiology, pathology, evolution

## Abstract

Les tumeurs conjonctives cutanées sont des tumeurs dermiques et/ou hypodermiques relativement fréquentes. Elles sont dominées par les tumeurs bénignes. A travers une série de 121 cas, nous avons étudié le profil épidémiologique, anatomopathologique et évolutif de ces tumeurs. C'est une étude rétrospective réalisée au service d'anatomie pathologique du CHU Mohammed VI de Marrakech entre 2004 et 2012. Il s'agit de 121 patients. La moyenne d’âge était de 36 ans (1-80ans). Le sex-ratio H/F était de 1,12. La tumeur avait un aspect nodulaire dans 90% des cas. Le membre inférieur était la localisation la plus fréquente (30,5%). L’étude anatomopathologique a porté sur un matériel biopsique dans 100% des cas. Soixante-neuf pour cent de ces tumeurs étaient bénignes. Elles étaient représentées essentiellement par les tumeurs vasculaires, suivies par les tumeurs fibreuses et fibro-histiocytaires. Trente et un pour cent des tumeurs étaient malignes. Il s'agissait essentiellement de tumeurs fibreuses et fibro-histiocytaires, suivies de tumeurs vasculaires. L’étude immunohistochimique était réalisée dans 2cas. Le traitement chirurgical était entrepris dans 73% des cas. L’évolution était précisée dans 19% des cas avec une évolution favorable dans 13% des cas. Un cas de décès et 2 cas de récidive étaient notés. Les tumeurs conjonctives cutanées bénignes sont de bon pronostic, mais posent un problème majeur de nosologie et de classification. D'autre part, la prise en charge diagnostique et thérapeutique ainsi que l’évaluation pronostique des sarcomes cutanés restent difficiles.

## Introduction

Les tumeurs conjonctives cutanées se développent à partir des éléments du tissu conjonctif propre cutané et de ses structures différenciées (vaisseaux sanguins, muscle, nerfs et graisse) [1]. Ainsi, elles prennent leur origine dans le derme et/ou l'hypoderme. Embryologiquement, elles dérivent principalement du mésoderme. Elles sont classées par l'OMS 2006 selon des bases histogénétiques [1, 2].

Les tumeurs conjonctives cutanées bénignes sont les plus fréquentes [2]. Elles rappellent sur le plan histologique le tissu normal et ont un faible pouvoir d'envahissement local ou de récidive après un traitement conservateur. Ces tumeurs posent un problème majeur de nosologie et de classification [1, 3]. Cependant, les sarcomes cutanés sont relativement rares et font partie des sarcomes des tissus mous superficiels. Leur diagnostic anatomopathologique est difficile et leur prise en charge, souvent problématique, est d'ordre multidisciplinaire [4]. La présentation clinique des tumeurs conjonctives cutanées les rend accessibles, pour la plupart d'entre elles à l'examen clinique et à la biopsie par le dermatologue. Ces tumeurs réalisent le plus souvent des tableaux anatomo-cliniques. D'où l'intérêt du contexte clinique (la présentation, les antécédents du patient et l’évolutivité de la lésion) [5, 6]. Le but de notre travail est de déterminer le profil épidémiologique, anatomopathologique et évolutif de ces tumeurs diagnostiquées dans notre service.

## Méthodes

Il s'agit d'une étude rétrospective descriptive portant sur 121 cas de tumeurs conjonctives cutanées colligées au service d'anatomie pathologique du CHU Mohammed VI de Marrakech sur une période de 9 ans allant du 01/01/2004 au 31/12/2012. Les données ont été recueillies à partir des registres et comptes rendus anatomo-pathologiques, des dossiers d'hospitalisation des patients aux services de dermatologie, de chirurgie plastique et d'oncologie.

## Résultats

Nous avons colligé 121 cas de tumeurs conjonctives cutanées sur une période de 9ans, ce qui correspond à un taux annuel moyen de 13,44 cas/an. Soixante-neuf pour cent des tumeurs étaient bénignes. L’âge moyen de nos patients était de 36ans avec des extrêmes allant de 1 à 80ans. La moyenne d’âge était respectivement de 37,5ans (1-70ans) et 49ans (13-80) pour les tumeurs bénignes et les tumeurs malignes. Le sex-ratio (H/F) était de 1,12. Un de nos patients était suivi pour une NF1. Quatre patients avaient développé des histiocytofibromes sur cicatrices de plaie superficielle (3cas) et de piqûre d'insecte (1cas). Un antécédent de tuberculose pulmonaire traitée et bien évoluée était retrouvé chez 2cas. Le premier avait un lipome et le 2ème avait un dermatofibrosarcome de Darrier Ferrand (DFSDF). Un patient avait un chancre syphilitique qui a bien évolué sous traitement. Il présentait une tumeur glomique. Aucun cas familial, d'infection virale, de brûlure, d'irradiation ou d'exposition professionnelle n’était retrouvé. Les malades consultaient pour des lésions nodulaires dans 90% des cas. La localisation prédominante était au niveau des membres. En effet, la localisation au membre inférieur était retrouvée dans 30,5% des cas. Des signes associés cutanés étaient retrouvés chez 4 malades. Il s'agissait de tâches café au lait chez 1 cas de NF1, de lentigines chez 2cas de neurofibrome et 2cas de Kaposi. Pour les signes extra cutanés, il s'agissait d'une atteinte de la muqueuse gingivale avec matité basithoracique et hépathomégalie chez 2cas de Kaposi. L'atteinte ganglionnaire était retrouvée chez 4 cas porteurs de sarcome de Kaposi. Sur le plan anatomopathologique, le matériel d’étude portait dans 65% des cas sur des biopsies exérèses et dans 35% des cas sur des biopsies simples. Le matériel d’étude était fixé au formol et inclus en paraffine. Une coloration à l′hématoxyline éosine était réalisée de façon systématique. L’étude immunohistochimique était réalisée dans 2cas de DFSDF où se posait le problème de diagnostic différentiel avec une origine neurogène ou musculaire lisse. Les anticorps utilisés étaient; la vimentine, le CD34, l'AML et la PS100. Les 2 tumeurs exprimaient la vimentine et le CD34 au niveau cytoplasmique.

**Figure 1:**
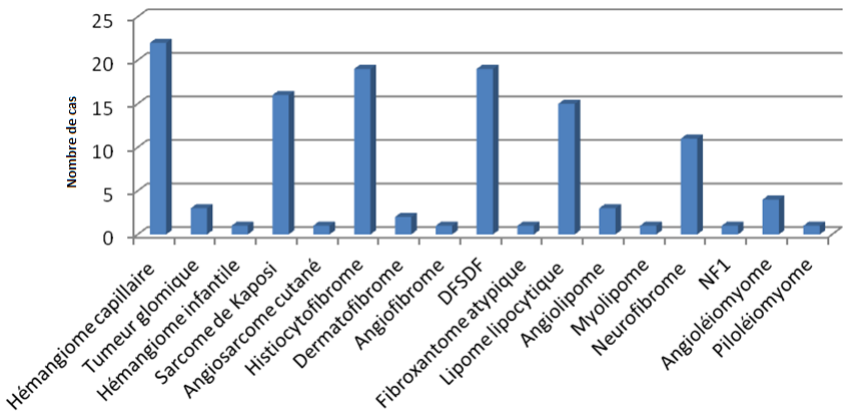
Répartition des patients selon le diagnostic anatomopathologique

Le diagnostic anatomopathologique est illustré par la Figure 1. Les tumeurs vasculaires étaient les plus fréquentes (35,5%) au même rang que les tumeurs fibreuses et fibro histiocytaires (34,7%). Elles étaient représentées essentiellement par des tumeurs bénignes (Tableau 1).

**Tableau 1:** Répartition des tumeurs selon leur origine histogénétique et leur nature bénigne ou maligne

	Nombre de cas	
	Tumeurs bénignes	Tumeurs malignes
Tumeurs vasculaires (35,5%)	26	17
Tumeurs fibreuses et fibro-histiocytaires (35%)	22	20
Tumeurs adipeuses (15,5%)	19	0
Tumeurs nerveuses (10%)	12	0
Tumeurs musculaires (4%)	5	0

L'exérèse chirurgicale était réalisée dans 73% des cas. Six patients atteints d'une maladie de Kaposi (8%) étaient traités par chimiothérapie. Un parmi eux avait subit une radiothérapie en complément thérapeutique. La guérison était notée chez 16 malades (13%). Il s'agissait d'un cas de neurofibrome, 5 cas de lipome, 6 cas d'histiocytofibrome et 4 cas d'hémangiome capillaire. Un cas de décès (Kaposi avec sérologie HIV positive) et 6 cas de récidive (un neurofibrome, un hémangiome capillaire d'exérèse incomplète et 4 cas de DFS dont les limites d'exérèse étaient lésionnelles) étaient notés. Le reste des malades ayant été perdus de vue avant ou au cours du traitement.

## Discussion

Les tumeurs conjonctives cutanées constituent un groupe hétérogène de tumeurs rapportées dans la littérature. Leur incidence réelle reste à préciser [5]. En dehors des tumeurs vasculaires dont 40% surviennent avant 20 ans, les tumeurs conjonctives cutanées bénignes s′observent à tout âge [2, 5]. Nos résultats rejoignent ceux de la littérature. Selon notre étude, l’âge moyen pour les tumeurs malignes était de 49ans (13-80ans). Le DFS était le sarcome cutané le plus fréquent. Le sex-ratio était de 1,12 avec une nette prédominance masculine. A notre connaissance, ces paramètres n'ont jamais été analysés de façon globale. La pathogénie de ces tumeurs est mal connue [1, 3, 5]. Cependant, des facteurs de risque d'ordre génétique, liés au terrain ou environnementaux sont rapportés dans la littérature [1, 5]. Dans notre étude, nous avons rapporté 4 cas d'histiocytofibromes dont 3 étaient survenus sur cicatrices de plaie superficielle et 1 sur cicatrice de piqûre d'insecte. Cependant, aucun cas familial, d'infection virale, de brûlure, d'irradiation ou d'exposition professionnelle n’était retrouvé.

Les nodules cutanés représentent le principal motif de consultation de ces tumeurs [7]. Dans notre étude, 90% des patients consultaient pour des nodules cutanés. La localisation prédominante au niveau des membres dans notre étude peut être expliquée par la prédominance des tumeurs fibrohistiocytaires ainsi que du sarcome de Kaposi [2, 5]. Les signes associés cutanés se rencontrent essentiellement dans le cadre de génodermatoses et de maladie de Kaposi [1, 4, 5]. Dans notre étude, il s'agissait essentiellement de tâches café au lait chez un cas de NF1, de lentigines chez 1cas de neurofibrome et 2cas de Kaposi. L'atteinte lymphatique, gastro intestinale et pulmonaire est souvent en rapport avec une maladie de Kaposi liée à une immunodépression acquise ou iatrogène [8]. Sur le plan anatomopathologique, ces tumeurs sont dominées par les tumeurs bénignes [1–3]. Dans notre étude, 69,5% de nos patients avaient des tumeurs bénignes et 30,5% étaient suivis pour des tumeurs malignes. Les tumeurs bénignes adipeuses et vasculaires sont les plus fréquentes [1, 2]. Nos résultats illustrés sur la figure 1 et le tableau II montrent que les tumeurs vasculaires étaient les plus fréquentes, suivies des tumeurs fibreuses. En effet, les lipomes ne sont réséqués dans notre contexte qu'en cas de gène esthétique.

Un point important à connaitre concernant ces tumeurs bénignes: D'une part, les neurofibromes et les lipomes ne figurent pas dans la classification des tumeurs des tissus mous cutanés de l'OMS 2006 [1]. Toutefois, ce sont des tumeurs qui naissent des structures nerveuses et adipeuses cutanées; éléments différenciés du tissu conjonctif de la peau. Ils sont alors soit décrits selon la classification des tumeurs de l'os et des parties molles de l'OMS 2002 [3], soit rapportés ailleurs dans des bouquins de dermatopathologie [8]. Par conséquent, nous les avons intégrés dans notre étude. Ce problème de classification émanerait de l'obscurité des mécanismes physiopathogéniques sous-jacents.

D'autre part, les tumeurs bénignes en général, continuent à poser un problème de nosologie. En effet, une même entité tumorale peut avoir plusieurs synonymes. Par exemple, l'hémangiome sinusoidal est appelé par certains auteurs hémangiome caverneux. De même, l'hyperplasie angiolymphoide avec éosinophilie (HALE) représente la même entité néoplasique que l'hémangiome épithélioide et la prolifération vasculaire atypique intraveineuse’ Cette terminologie multiple est en fait sorte de confusion et peut influer directement sur la qualité de la prise en charge.

Selon notre revue de la littérature, aucune étude n'a comparé la fréquence des différents sarcomes cutanés. Les tumeurs malignes les plus fréquentes dans notre série étaient celles d'origine fibreuse, suivies par les tumeurs vasculaires. La tumeur fibreuse maligne la plus fréquente était le DFSDF, suivi par le sarcome de Kaposi; tumeur vasculaire maligne la plus fréquente. La chirurgie reste le traitement principal des tumeurs conjonctives de la peau. Celles-ci étant en général facilement résécables (à l'exception du DFSDF et de l'angiosarcome) [9]. Dans notre série, une exérèse chirurgicale était réalisée chez 73% des patients. Pour certaines tumeurs malignes, le pronostic dépend essentiellement de la profondeur de la lésion, les lésions dermiques pures étant de petite taille et d'excellent pronostic [10, 11].

## Conclusion

Malgré leur rareté, les tumeurs conjonctives cutanées constituent un spectre large, hétérogène et complexe dans le paysage de la pathologie tumorale cutanée. Cette étude, quoi que limitée à notre région nous a aidé à dresser le profil épidémiologique, anatomopathologique et évolutif de ces tumeurs. Par ailleurs, elle nous a permis de soulever le double problème des tumeurs bénignes; de classification et de nosologie. Cependant, des études multicentriques, à l’échelle nationale et internationale s'avèrent nécessaires. Ceci inciterait à fournir des efforts pour pouvoir parler un langage commun et améliorer la prise en charge des patients. Les sarcomes cutanés, de leurs côté, peuvent poser des problèmes de diagnostic anatomopathologique, d’évaluation pronostique et de stratégie thérapeutique. En effet, des études plus approfondies et concentrées sur la pathologie tumorale conjonctive cutanée indépendemment de la pathologie tumorale générale des parties molles, sont impératives. Celles-ci doivent également tenir compte du comportement et de la signature biologiques de ces tumeurs afin d'aboutir à de nouveaux moyens thérapeutiques, voire préventifs.
